# Associations of *NR5A2* Gene Polymorphisms with the Clinicopathological Characteristics and Survival of Gastric Cancer

**DOI:** 10.3390/ijms151222902

**Published:** 2014-12-10

**Authors:** Xunlei Zhang, Dongying Gu, Mulong Du, Meilin Wang, Chunxiang Cao, Lili Shen, Meng Kuang, Yongfei Tan, Xinying Huo, Weida Gong, Zhi Xu, Jinfei Chen, Zhengdong Zhang, Cuiju Tang

**Affiliations:** 1 Department of Oncology, Nanjing First Hospital, Nanjing Medical University, 68 Changle Road, Nanjing 210006, China; E-Mails: zhangxunlei1@sina.com (X.Z.); gu_dongying@sina.com (D.G.); chunxiang198722@163.com (C.C.); shenlilimao2012@163.com (L.S.); jhcxy1225@126.com (M.K.); huoxinying2008@126.com (X.H.); michelle.xuzhi@gmail.com (Z.X.); 2 Department of Oncology, Nantong Tumor Hospital, Nantong 226300, China; 3 Department of Environmental Genomics, Jiangsu Key Laboratory of Cancer Biomarkers, Prevention and Treatment, Cancer Center, Nanjing Medical University, Nanjing 211166, China; E-Mails: dumulong@163.com (M.D.); meilin_w@163.com (M.W.); 4 Department of Genetic Toxicology, the Key Laboratory of Modern Toxicology of Ministry of Education, School of Public Health, Nanjing Medical University, Nanjing 211166, China; 5 Department of Surgery, Yixing People’s Hospital, Yixing 214200, China; E-Mail: shelly37319@126.com; 6 Department of General Surgery, Yixing Tumor Hospital, Yixing 214200, China; E-Mail: gongweida2010@gmail.com

**Keywords:** *NR5A2*, rs3790843, rs3790844, single nucleotide polymorphism, gastric cancer

## Abstract

The orphan nuclear receptor (NR5A2), which belongs to the NR5A subfamily of nuclear receptors, is expressed in developing and adult tissues of endodermal origin, and can contribute to the development of several cancers through regulating cell proliferation. *NR5A2* (rs3790843 and rs3790844) single nucleotide polymorphisms (SNPs) genotyping were examined in DNA samples, extracted from paraffin-embedded cancer tissue. Clinicopathologic and follow-up data were collected from 944 patients with gastric cancer (GC). Associations of the 2 SNPs with the progression and prognosis in gastric cancer patients were analyzed using the SPSS version 18.0. We found that *NR5A2* rs3790843 polymorphism was significantly associated with the risk of GC which had regional lymph node metastasis (*p* = 0.044) or distant metastasis (*p* = 0.020). Our results also indicated that rs3790844 polymorphism was associated with the increased overall survival (OS) of GC patients in the dominant model (GG *vs.* GA/AA, HR (hazard ratio) = 0.823, 95% CI (confidence interval) = 0.679–0.997), suggesting a potential protective role of the variant A allele. Additionally, in the stratified analysis, both *NR5A2* rs3790843 and rs3790844 polymorphism were associated with significantly lower risk of death in the groups of female, tumor size >5 cm in a dominant model. Our results represent the first demonstration that the *NR5A2* rs3790844 polymorphism is associated with increased OS of GC patients in the dominant model, and similar results were found among the female group and tumor size >5 cm group for *NR5A2* rs3790843 polymorphism. Further validation in other larger studies with different ethnic populations and functional evaluations are needed.

## 1. Introduction

Gastric cancer (GC), as the third leading cause of cancer death in men and the fifth in women, remains one of the major public health problems worldwide [1]. About two-thirds of the cases occur in developing countries and 42% in China alone, while the estimated high-risk areas in developed countries include Eastern Europe, and parts of Central and South America [2]. In recent decades, its incidence rate has declined, and remarkable progress has been achieved in comprehensive treatment strategies of combined therapy. However, the prognosis for patients still remains poor, with 5-year overall survival rates of 30% [3]. The development of gastric cancer is a multi-step, sequential process, which initiates from chronic gastritis, atrophy, intestinal metaplasia, dysplasia, and finally malignant transformation to invasive gastric cancer [4]. Moreover, multiple genetic and epigenetic alterations are implicated in the multi-step process of human stomach carcinogenesis and development [5]. Therefore, discovery of these biomarkers could be helpful in the improvement of early diagnosis, screening of high-risk individuals, as well as patient care [6,7]. In recent years, increased studies have focused on the detection of genetic variants that could play roles in the development, progression and prognoses of gastric cancer [8].

The orphan nuclear receptor (NR5A2), also known as liver receptor homologue-1 (LRH-1) and fetoprotein transcription factor (FTF), belongs to the NR5A or FTZ-F1 subfamily of nuclear receptors. NR5A2 is expressed in developing and adult tissues of endodermal origin, including liver, pancreas, intestine and the ovary [9,10]. Functionally, NR5A2 has been implicated in the regulation of bile acid and cholesterol homeostasis [9,10] and the regulation of inflammatory responses in the liver and gut [11]. Aromatase is an *NR5A2* target gene, which catalyses the conversion of androgens (testosterone and primarily androstenedione) to oestrogens. *NR5A2* may aid breast cancer progression in postmenopausal women by promoting local oestrogen biosynthesis [12,13,14]. The expression of *NR5A2* is also elevated in pancreatic cancer and promotes pancreatic cancer cell growth through stimulation of *cyclin D1*, *cyclin E1 and c-Myc* [15]. A recent genome-wide association study (GWAS) identified rs3790844 and rs3790843, located in the first intron of *NR5A*2 in 1q32.11, as associated with pancreatic cancer susceptibility [16]. In the gastrointestinal tract, *NR5A2* has been shown to participate in intestinal cell renewal [17] and is expressed in the stomach epithelium [18]. Therefore, we conducted this study to examine whether *NR5A2* rs3790844 and rs3790843 polymorphisms are associated with clinical outcomes of gastric cancer. The two SNPs may serve as potential molecular prognostic markers for gastric cancer, which will promote further defined sub-populations at higher risk of the disease. Consequently, these sub-populations may require more rigorous treatment and postoperative follow up.

## 2. Results

### 2.1. Associations between Clinicopathological Variables and Overall Survival

The patients’ characteristics and clinical information are summarized in Table 1. In the follow-up period of 119 months, 442 patients died. There were 727 males (77.0%) and 217 females (23.0%), with a median age of 62 years ranging from 28 to 83 years. Clinicopathological characteristics including tumor size, depth of invasion, lymph node metastasis, distant metastasis, Tumor, Node and Metastasis (TNM) stage and Lauren classification were significantly associated with survival time (log-rank *p* < 0.05). Specifically, patients with tumor size >5 cm (median survival time (MST), 48 months) had a 42% significantly higher risk of death (HR (hazard ratio) = 1.42, 95% CI (confidence interval) = 1.178–1.716), compared with those with tumor size <5 cm (MST, 98 months), and the patients with lymph node metastasis or distant metastasis had significantly higher risk of death than those patients without lymph node metastasis or distant metastasis (log-rank *p* < 0.05). In addition, as the depth of invasion and TNM stage increased, the risk of death for gastric cancer showed a significant increase in a dose-response manner (log-rank *p* < 0.05).

**Table 1:** Associations between clinicopathological variables and overall survival.

Variable	Patients, *n* = 944	MST (Months)	Log-Rank *p*	HR (95% CI) ^d^
**Age (years)**				
≤60	442	88	0.239	1.000
>60	502	59		1.118 (0.927–1.349)
**Sex**				
Male	727	70	0.536	1.000
Female	217	63		1.071 (0.860–1.335)
**Tumor Size**				
≤5 cm	581	98	<0.001	1.000
>5 cm	363	48		1.422 (1.178–1.716)
**Location**				
Non-Cardia	626	78	0.253	1.000
Cardia	318	63		0.891 (0.730–1.088)
**Histological Types ^a^**				
Intestinal	370	74	0.436	1.000
Diffuse	497	60		1.069 (0.918–1.244)
**Differentiation ^a^**				
Well to Moderate	305	80	0.518	1.000
Poorly	493	59		1.165 (0.942–1.443)
Mucinous/Signet-Ring Cell	69	62		1.199 (0.825–1.742)
**Lauren ^a^**				
1	399	76	<0.001	1.000
2	541	50		1.467 (1.211–1.776)
**Depth of Invasion ^b^**				
T1	182	84	<0.001	1.000
T2	138	78		0.540 (0.408–0.714)
T3	8	70		0.800 (0.608–1.054)
T4	594	51		1.008 (0.416–2.442)
**Lymph Node Metastasis ^c^**				
N0	378	81	<0.001	1.000
N1/N2/N3	566	43		1.814 (1.480–2.222)
**Distant Metastasis**				
M0	886	74	0.004	1.000
M1	58	26		1.646 (1.165–2.326)
**TNM Stage**				
I	250	83	<0.001	1.000
II	203	88		1.231 (0.909–1.666)
III	458	41		1.949 (1.524–2.492)
IV	25	44		2.046 (1.163–3.600)
**Chemotherapy**				
No	638	61	0.684	1.000
Yes	306	69		1.043 (0.852–1.276)

Mean survival time was presented when the median survival time could not be measured. TNM, Tumor, Node and Metastasis; MST, median survival time; HR, hazard ratio; CI, confidence interval; AJCC, American Joint Commission on Cancer. ^a^: Partial data were not available, and statistics were based on available data; ^b^: Invaded depth of tumor was classified according to the criteria of AJCC 7th; ^c^: Lymph nodes were staged according to tumor node metastasis classification of the 7th edition of AJCC in which the number of lymph nodes with a metastasis of 1–2, 3–6, and ≥7 were classified as N1, N2, and N3, respectively; ^d^: Adjusted by age and sex.

### 2.2. Association Analyses of NR5A2 rs3790843 and rs3790844 Genotypes with Clinicopathological Features

Among 944 specimens from patients with gastric cancer, *NR5A2* rs3790843 was successfully genotyped in 907 specimens, and *NR5A2* rs3790844 was successfully genotyped in 912 specimens. The frequency of each *NR5A2* rs3790843 genotypes was 45.6% (414 specimens) for the TT variant, 45.2% (410 specimens) for the CT variant, and 9.2% (83 specimens) for the CC variant. Table 2 indicates that the risk of GC for the CT and CC variants compared with that for the TT variant in *NR5A2* rs3790843 was associated significantly with regional lymph node metastasis (*p* = 0.044) and distant metastasis (*p* = 0.020) but not with age, sex, tumor size or location, histological type, differentiation, depth of invasion, chemotherapy history, American Joint Committee on Cancer staging, or Lauren classification. In reference to *NR5A2* rs3790844, the GG, GA, and AA genotypes were identified in 399 specimens (43.8%), 416 specimens (45.6%), and 97 specimens (10.6%), respectively. Compared with the GG genotype, the GA/AA genotypes were not associated with any clinicopathologic data.

**Table 2:** Association analyses of *NR5A2* rs3790843 and rs3790844 genotypes with clinicopathological features.

Variable		rs3790843 (*n* = 907)			rs3790844 (*n* = 912)		
		TT	TC/CC	*p*	GG	GA/AA	*p*
**Age (years)**	≤60	192	237	0.610	182	249	0.380
	>60	222	256		217	264	
**Sex**	Male	321	375	0.601	311	391	0.539
	Female	93	118		88	122	
**Location**	Non-Cardia Cancer	279	320	0.432	264	338	0.930
	Cardia Cancer	135	173		135	175	
**Tumor Size**	≤5 cm	261	302	0.581	243	324	0.486
	>5 cm	153	191		156	189	
**Lymph Node Metastasis ^a^**	N0	180	182	0.044	168	197	0.257
	N1/N2/N3	234	311		231	316	
**Distant Metastasis**	M0	398	456	0.020	382	478	0.098
	M1	16	37		9	35	
**Histological Types ^b^**	Intestinal	168	187	0.273	156	205	0.541
	Diffuse	207	270		206	270	
**Differentiation ^b^**	Well to Moderate	137	155	0.556	127	170	0.687
	Poorly	208	267		205	270	
	Mucinous/Signet-Ring Cell	30	35		30	35	
**Lauren ^b^**	1	182	199	0.253	169	218	0.450
	2	229	293		227	294	
**Chemotherapy**	No	275	333	0.721	266	348	0.709
	Yes	139	160		133	165	
**Depth of Invasion ^c^**	T1	86	93	0.854	76	101	0.929
	T2	60	69		59	71	
	T3	3	3		2	4	
	T4	257	320		254	326	
**TNM Stage**	I	120	118	0.369	111	128	0.533
	II	83	111		78	119	
	III	198	248		198	250	
	IV	10	13		9	13	

HR, hazard ratio; CI, confidence interval; AJCC, American Joint Commission on Cancer; TNM, Tumor, Node and Metastasis. ^a^: Lymph nodes were staged according to tumor node metastasis classification of the 7th edition of AJCC in which the number of lymph nodes with a metastasis of 1–2, 3–6, and ≥7 were classified as N1, N2, and N3, respectively; ^b^: Partial data were not available, and statistics were based on available data; ^c^: Invaded depth of tumor was classified according to the criteria of AJCC 7th.

The distribution of genotypes in the population was consistent with Hardy–Weinberg equilibrium (*p* = 0.20 for rs3790843 SNP, *p* = 0.46 for rs3790844 SNP), while strong linkage disequilibrium (LD) exists between alleles of the two loci with the following *R*_2_ values: rs3790843/rs3790844 = 0.798. Haplotype analyses were performed for all patients using the SHEsis software (http://analysis. bio-x.cn), and the six possible haplotype frequencies of the two SNPs are shown in Table 3. According to the results, the TTGG and CTGA haplotypes are the most common and represent 41.5% and 40.8%, respectively, in all groups. After haplotype analyses, however, no significant differences in these haplotype frequencies were found in any group.

**Table 3:** Haplotype analyses of *NR5A2* rs3790843 and rs3790844 genotypes.

Haplotype	Frequencies	Patients/Deaths	HR (95% CI) ^a^
TTGG	0.415	379/184	1.00
CTGA	0.408	373/170	0.90 (0.73–1.10)
CCAA	0.079	72/30	0.79 (0.54–1.17)
TTGA	0.036	33//11	0.54 (0.30–1.00)
CTAA	0.024	22//6	0.47 (0.21–1.07)
CTGG	0.015	14//10	1.55 (0.81–2.94)

^a^: Adjusted by age and sex.

### 2.3. Associations of NR5A2 rs3790843 and rs3790844 with Clinical Outcomes of OS

**Figure 1:**
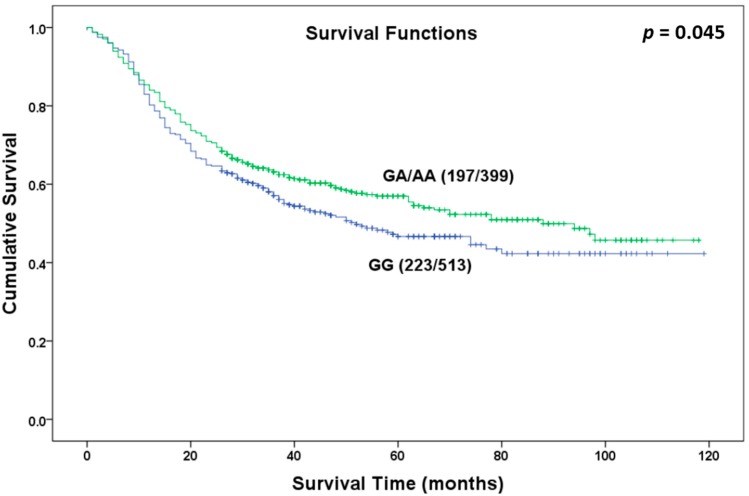
Overall survival curve in relation to *NR5A2* rs3790844 polymorphism in patients with gastric cancer in dominant model.

The Kaplan-Meier survival analysis and Cox proportional hazard models were used to assess the prognostic effect of *NR5A2* rs3790843 and rs3790844 on GC patients in different genetic models (Table 4). We found rs3790844 polymorphism was associated with the OS of GC patients in the dominant model (GG *vs.* GA/AA, log-rank *p* = 0.045, Figure 1). Patients with the GA or AA genotype (MST 88 months) had a 17.7% lower risk of death than that of patients with the GG genotype (MST 52 months) (HR = 0.823, 95% CI = 0.679–0.997), suggesting a potential protective role of the variant A allele. No significant associations were observed between the rs3790843 genotypes and OS of GC patients in any genetic models. We further evaluated the associations by stratified analysis of variants of clinicopathological features (Table 5). In the dominant model, *NR5A2* rs3790844 polymorphism was associated with significantly lower risk of death in the groups of patients with lymph node metastasis, no distant metastasis, diffuse type and no chemotherapy history (log-rank *p* < 0.05). Additionally, both *NR5A2* rs3790843 and rs3790844 polymorphism were associated with significantly better prognosis among the female patients group and tumor size >5 cm group in a dominant model. Cox stepwise regression analysis was conducted to evaluate the independent effect of clinicopathological variables, rs3790843 and rs3790844 SNP on the OS of the patients with GC. As shown in Table 6, two variables (lymph node metastasis and *NR5A2* rs3790844) were included in the regression model by stepwise selection of the covariant variables and rs3790844 SNP was shown to be an independent protective factor for GC with a 21.6% decreased risk (HR = 0.784, 95% CI = 0.646–0.951, *p* = 0.014).

**Table 4:** Associations of *NR5A2* rs3790843 and rs3790844 with clinical outcomes of overall survival.

Genetic Model	Genotypes	Patients	Deaths	MST (Months)	Log-Rank *p*	HR (95%CI) ^a^
**rs3790843**						
Codominant Model	TT	414	196	59	0.668	1.000
	CT	410	187	70		0.934 (0.765–1.142)
	CC	83	36	88		0.872 (0.611–1.244)
Dominant Model	TT	414	196	59	0.415	1.000
	CT or CC	493	223	70		0.924 (0.762–1.119)
Recessive Model	TT or CT	824	383	70	0.551	1.000
	CC	83	36	88		0.902 (0.641–1.269)
**rs3790844**						
Codominant Model	GG	399	197	52	0.075	1.000
	GA	416	186	78		0.853 (0.698–1.043)
	AA	97	37	98		0.699 (0.492–0.993)
Dominant Model	GG	399	197	52	0.045	1.000
	GA or AA	513	223	88		0.823 (0.679–0.997)
Recessive Model	GG or GA	815	383	65	0.103	1.000
	AA	97	37	98		0.757 (0.540–1.061)

MST, median survival time; HR, hazard ratio; CI, confidence interval. Mean survival time was presented when the median survival time could not be measured. ^a^: Adjusted by age and sex.

## 3. Discussion

In the present study, we investigated the effects of two SNPs (rs3790843 and rs3790844) of the *NR5A2* gene on the progression and survival of GC in Chinese populations. We found that *NR5A2* rs3790843 polymorphism was significantly associated with the risk of GC when compared with regional lymph node metastasis and distant metastasis. Our results also indicated that rs3790844 polymorphism was associated with the increased OS of GC patients in the dominant model, suggesting a potential protective role of the variant A allele. No significant associations were observed between the rs3790843 genotypes and OS of GC patients in any genetic models. Additionally, in the stratified analysis, *NR5A2* rs3790844 polymorphism was associated with significantly lower risk of death in the groups of female, tumor size >5 cm, lymph node metastasis, no distant metastasis, diffuse type and no chemotherapy history in the dominant model. Similar results were found among the female patients group and tumor size >5 cm group for the *NR5A2* rs3790843 polymorphism in a dominant model.

Nuclear receptor 5 subtype A2 (NR5A2), also known as Liver receptor homolog-1 (LRH-1), is a member of the orphan family of nuclear receptors that participates in a wide range of developmental processes [9,10]. Functionally, NR5A2 has been implicated in the regulation of bile acid and cholesterol homeostasis, and is expressed in developing and adult tissues of endodermal origin, including liver, pancreas, intestine and the ovary [9,10]. *NR5A2* was first isolated as a transcriptional activator of the alpha-fetoprotein (*AFP*) gene, which encodes a factor that plays a crucial rolein hepatic specification [19]. Further, *NR5A2* expression is elevated in pancreatic cancer andpromotes pancreatic cancer cell growth through stimulation of *cyclin D1*, *cyclin E1 and c-Myc* [15], while genome-wide association studies implicate mutations in the *NR5A2* gene in pancreatic ductal adenocarcinoma [16]. In the gastrointestinal tract, *NR5A2* has been shown to participate in intestinal cell renewal [17] and is expressed in the stomach epithelium [18]. However, it seemed no study focused on the association between *NR5A2* and the progression and prognoses of gastric cancer. Thus, this study was conducted, and it indicates that the *NR5A2* polymorphism is significantly associated with the development and overall survival of gastric cancer.

In the gastrointestinal tract, *NR5A2* regulates proliferation through at least two different mechanisms: direct activation of the *cyclin E1* promoter and, in cooperation with *β-catenin*, induction of *cyclin D1* and *c-Myc* transcription [20]. A previous study showed that *NR5A2*, which interacts with *β-catenin*, induces cell proliferation through the concomitant induction of *cyclin D1* and *E1*, which are downstream *NR5A2* effectors whose induction may account for the diminished threshold for G/S transition [20]. Whereas *β-catenin* coactivates *NR5A2* on the *cyclin E1* promoter, *NR5A2* acts as a potent tissue-restricted coactivator of *β-catenin* on the *cyclin D1* promoter.

**Table 5:** Stratified analysis of rs3790843 and rs3790844 polymorphism among GC patients.

	rs3790843				rs3790844			
	TT	TC/CC	HR (95% CI) ^e^	*p*	GG	GA/AA	HR (95% CI) ^e^	*p*
**Age (years)**								
≤60	88/192	108/237	0.977 (0.737–1.294)	0.869	90/182	107/249	0.806 (0.609–1.067)	0.132
>60	108/222	115/256	0.873 (0.672–1.136)	0.313	107/217	116/264	0.837 (0.644–1.089)	0.185
**Sex**								
Male	144/321	175/375	1.044 (0.838–1.302)	0.700	149/311	172/391	0.877 (0.705–1.093)	0.243
Female	52/93	48/118	0.619 (0.417–0.916)	0.017	48/88	51/122	0.660 (0.445–0.981)	0.040
**Location**								
Non–CardiaCancer	133/279	148/320	0.948 (0.750–1.198)	0.652	133/264	148/338	0.810 (0.641–1.024)	0.077
CardiaCancer	63/135	75/173	0.861 (0.615–1.204)	0.381	64/135	75/175	0.840 (0.601–1.174)	0.308
**Tumor Size**								
≤5 cm	108/261	127/302	1.044 (0.807–1.349)	0.745	106/243	132/324	0.927 (0.718–1.197)	0.559
>5 cm	88/153	96/191	0.747 (0.558–0.999)	0.049	91/156	91/189	0.693 (0.517–0.928)	0.014
**Lymph Node Metastasis ^a^**								
N0	64/180	60/182	0.947 (0.666–1.347)	0.763	63/168	63/197	0.865 (0.610–1.226)	0.414
N1/N2/N3	132/234	163/311	0.829 (0.659–1.043)	0.109	134/231	160/316	0.744 (0.591–0.937)	0.012
**Distant Metastasis**								
M0	188/398	200/456	0.886 (0.726–1.081)	0.232	188/382	202/478	0.793 (0.650–0.968)	0.022
M1	8/16	23/37	1.340 (0.599–2.998)	0.476	7/9	21/35	1.189 (0.544–2.599)	0.664
**Histological Types ^b^**								
Intestinal	71/168	80/187	1.041 (0.756–1.434)	0.805	69/156	84/205	0.952 (0.692–1.309)	0.762
Diffuse	108/207	127/270	0.826 (0.639–1.068)	0.145	111/206	123/270	0.735 (0.568–0.950)	0.019
**Differentiation ^b^**								
Well to Moderate	57/137	67/155	1.099 (0.772–1.565)	0.601	56/127	69/170	0.962 (0.676–1.368)	0.828
Poorly	104/208	126/267	0.882 (0.680–1.144)	0.344	105/205	125/270	0.822 (0.634–1.067)	0.140
Others ^c^	18/30	14/35	0.522 (0.259–1.056)	0.07	19/30	13/35	0.397 (0.195–0.808)	0.011
**Lauren ^b^**								
1	69/182	78/199	1.049 (0.759–1.451)	0.771	69/169	80/218	0.899 (0.652–1.241)	0.655
2	125/229	144/293	0.824 (0.648–1.047)	0.113	126/227	142/294	0.763 (0.600–0.970)	0.027
**Chemotherapy**								
No	132/275	151/333	0.908 (0.719–1.147)	0.416	136/266	149/348	0.767 (0.608–0.968)	0.025
Yes	64/139	72/160	0.957 (0.683–1.340)	0.799	61/133	74/165	0.953 (0.679–1.338)	0.783
**Depth of Invasion ^d^**								
T1	25/86	34/93	1.297 (0.774–2.173)	0.324	24/76	33/101	1.051 (0.621–1.779)	0.852
T2	31/60	24/69	0.669 (0.393–1.141)	0.140	31/59	25/71	0.648 (0.382–1.099)	0.107
T3	1/3	2/3	1.405 (0.125–15.838)	0.782	1/2	2/4	0.809 (0.071–9.157)	0.864
T4	134/257	159/320	0.880 (0.699–1.108)	0.276	135/254	157/326	0.809 (0.642–1.019)	0.072
**TNM Stage**								
I	40/120	39/118	0.992 (0.638–1.542)	0.972	40/111	40/128	0.862 (0.556–1.336)	0.506
II	38/83	38/111	0.743 (0.474–1.166)	0.196	37/78	41/119	0.711 (0.456–1.109)	0.132
III	111/198	135/248	0.861 (0.670–1.108)	0.245	113/198	132/250	0.800 (0.622–1.029)	0.082
IV	4/10	8/13	1.855 (0.557–6.171)	0.314	4/9	7/13	1.257 (0.368–4.302)	0.715

HR, hazard ratio; CI, confidence interval; AJCC, American Joint Commission on Cancer; TNM, Tumor, Node and Metastasis. ^a^: Lymph nodes were staged according to tumor node metastasis classification of the 7th edition of AJCC in which the number of lymph nodes with a metastasis of 1–2, 3–6, and ≥7 were classified as N1, N2, and N3, respectively; ^b^: Partial data were not available, and statistics were based on available data; ^c^: Others: mucinous carcinoma and Signet-ring cell carcinoma; ^d^: Invaded depth of tumor was classified according to the criteria of AJCC 7th; ^e^: Adjusted by age and sex.

**Table 6:** Stepwise Cox regression analysis on the survival of GC.

Variables	β	SE	HR ^b^	95% CI	*p* Value
**Age ^a^**	0.067	0.100	1.070	(0.878–1.302)	0.503
**Sex**	0.04	0.118	1.041	(0.825–1.313)	0.743
**Lymph Node Metastasis**	0.567	0.112	1.763	(1.414–2.198)	<0.001
rs3790844 (GG *vs.* GA/AA)	−0.243	0.099	0.784	(0.646–0.951)	0.014
rs3790843 (TT *vs.* CT/CC)	−0.152	0.101	0.859	(0.705–1.046)	0.131

β, regression coefficient; SE, standard error; HR, hazard ratio; CI, confidence interval. ^a^: Age was included as a continuous variable in the Cox stepwise regression analysis; ^b^: Adjusted by age and sex.

Tumor growth is the result of uncontrolled cell proliferation or a defective cell death program. For the proliferation of cells, the phosphorylation of pRb by *cyclin–CDK* complexes to release the transcription factor E2F is an essential step. The *cyclin D1*, *cyclin E* and *c-Myc* are important parts of expression peaks at the G1–S transition, and then decreases as cells proceed through the S phase [21,22,23]. The G1–S transition is the major regulation point of the cell cycle, and during the G1–S transition, alterations in cell cycle regulators lead to the deregulation of the cell cycle, which can cause unbridled cell division, contributing to cancer development. Previously, overexpression of *cyclin E* was demonstrated in many tumors and correlated with prognosis [24,25,26,27] including gastric cancer [28]. Wang *et al.* [29] found that the mRNA expression of NR5A2 was significantly upregulated in gastric cancer, as compared with self-paired normal control. In addition, overexpression of NR5A2 was shown to promote the proliferation of gastric adenocarcinoma SGC-7901 cells via induction of *cyclin E1*, which may lead to the tumorigenesis of gastric cancer [29]. Botrugno *et al.* [20] found that *cyclin E* was a direct *NR5A2* target gene, which could partly explain the mechanism of *NR5A2* polymorphism in the progression and prognoses of gastric cancer. On the other hand, *β-catenin* has been proposed to act as a docking protein that assembles both general and specific factors required for the activation of target genes. Botrugno *et al.* also found that *NR5A2* could act as a coactivator for *β-Catenin/Tcf4* to drive the expression of *cyclin D1* and other *β-catenin/Tcf* target genes, such as *c-Myc* [30,31,32]. As reported, *cyclin D1* is a proto-oncogene that belongs to the family of G1 cyclins, and plays an important role in cell cycle G1 to S transition by binding its partners cyclin dependent kinase 4 and 6 to phosphorylate and inactivate the Rb protein [33]. In addition, *cyclin D1* over-expression is usually an early event in carcinogenesis and a prognostic indicator associated with poor survival in cancers [34]. Given above-mentioned evidences, *NR5A2* polymorphism could also affect the carcinogenesis and prognoses of cancer through acting as a coactivator for *β-catenin* on the *cyclin D1* promoter.

Interestingly, in the stratified analyses by sex, our results indicated that both the *NR5A2* rs3790843 and rs3790844 polymorphisms were associated with significantly better prognosis in the female patient group. *NR5A2* is a direct estrogen receptor α (ERα) target gene [35,36], its expression correlates with ERα in breast tumours [37] and it promotes breast cancer proliferation and invasion [38]. Previous study showed that *NR5A2* is an important regulator of ERα target genes and it shares many binding sites with ERα. Importantly, at shared sites, *NR5A2* promotes ERα recruitment and *vice versa*, ERα stimulates *NR5A2* recruitment [39]. As a result, in the female patients with more ERα, more *NR5A2* polymorphisms, which act as a protective factor, contribute to a better prognosis.

Taken together, the presented findings of the potential involvement of the *NR5A2* gene in anti-tumorigenesis prompts us to further characterize its structure, biological function and interaction with other partners by *in vitro* or *in vivo* studies. However, some limitations of the present study should be addressed. First, Helicobacter pylorus, a known crucial factor in gastric carcinogenesis, was not considered due to the lack of related follow-up information. Second, only two SNPs in *NR5A*2 are evaluated, and it is possible that some other important SNPs are neglected or the observed associations may be due to other polymorphisms in linkage disequilibrium with the rs3790843 and rs3790844 polymorphisms. Finally, for validation of the genotype–phenotype relationship, further investigation is underway to clarify the association between rs3790843 and rs3790844 polymorphisms and expression levels of NR5A2 protein in gastric cancer tissues, and will be reported separately.

## 4. Experimental Section

### 4.1. Study Subjects

A retrospective cohort of 944 patients with gastric cancer who underwent a surgical resection at the Yixing People’s Hospital (Yixing, Jiangsu Province, China) between 1999 and 2006, who had a median follow-up of 35 months (range, 0–119 months) were recruited for this study [40]. The demographic features and clinicopathologic data were summarized in Table 1. All patients were diagnosed with gastric carcinoma histopathologically. None had received neoadjuvant radiochemotherapy or postoperative radiotherapy, and some had received adjuvant chemotherapy. Formalin-fixed and paraffin-embedded blocks of these patients were collected from the department of pathology of this hospital. The samples used for genotyping were reviewed and classified by two independent pathologists. Death dates were confirmed via review of death certificates of inpatient and outpatient records or obtained from patients’ families through follow-up telephone calls. The study protocol was approved by the Institutional Review Board of Nanjing Medical University (Nanjing, China), and all patients provided written informed consent on the use of paraffin specimens for gene polymorphism analyses.

### 4.2. Genotyping

Genomic DNA was extracted from tumor specimens using proteinase K digestion, followed by isopropanol extraction and ethanol precipitation. The *NR5A2* (rs3790844 and rs3790843) SNPs were examined by multiplex snapshot technology using an ABI fluorescence-based assay allelic discrimination method (Applied Biosystems, Foster City, CA, USA), which has been described indetail in a previous study [41]. The primers were designed to anneal immediately adjacent to the nucleotideat the mutation site: rs3790844: forward, 5'-TTCCGTGTGGAAACACAGGTCA-3'; reverse, 5'-TCGACTGGAGCCCAAGATCA-3'; rs3790843: forward, 5'-TCTTTGCCCCGATGAGTTCG-3'; reverse, 5'-TCCCAGATGCTCTGGTGCAG-3'. The primers for extension were as follows: rs3790844, 5'-TTTTTTTTTTTTT…TTTTTTTTTTTTTTTTTGGAAACACAGGTCACTAAAACTGG-3'; and rs3790843, 5'-TCTGGTGCAGCCGAAGTAG-3'. The snapshot products were analyzed by using an ABI 3130 genetic analyzer (Applied Biosystems) and the genotypes were determined by GeneMapper Analysis Software version 4 (Applied Biosystems). Genotype analysis was performed by two investigators blinded to the survival end points. Genotyping was validated by sequencing a randomly selected 10% of samples, and the results were 100% concordant. However, 37 cases of rs3790843 and 32 cases of rs3790844 failed in genotyping because of poor DNA quality, which were excluded in further analysis. Finally, 907 cases of rs3790843 and 912 cases of rs3790844 were included in the analysis.

### 4.3. Statistical Methods

The associations of each genotype or combinations of genotypes with clinicopathologic features were compared using the Pearson chi-square test for categorical variables and the Student’s *t*-test for continuous data. Associations of genetic variants and clinicopathological features with the overall survival (OS) were estimated using Kaplan-Meier method and comparisons between different groups of patients were performed with the log-rank test. Univariate or multivariate Cox proportional hazard models was used to estimate the adjusted HR and their 95% CI. Moreover, Cox stepwise regression analysis was conducted to assess the independent impacts of SNP or clinicopathological features on the overall survival after adjusting for other covariates, with a significance level of *p* < 0.05 for entering and *p* > 0.10 for removing the respective explanatory variables. All tests were two-sided and *p* < 0.05 was considered statistically significant. All the statistical analyses were carried out using SPSS version 16.0 for Windows (SPSS Inc., Chicago, IL, USA).

## 5. Conclusions

In conclusion, our results represent the first demonstration that *NR5A2* rs3790844 polymorphism was associated with the increased OS of GC patients in the dominant model, and similar results were found among the female patients group and tumor size >5 cm group for the *NR5A2* rs3790843 polymorphism, suggesting that the mutant alleles may serve as suitable markers for predicting the survival of gastric cancer patients, especially in a Chinese population. Large population-based prospective studies with ethnically diverse populations are warranted to verify these findings.
